# Regression adjustment for causal inference

**DOI:** 10.1136/bmjmed-2023-000816

**Published:** 2025-09-16

**Authors:** Frederick Ho

**Affiliations:** 1 School of Health and Wellbeing, University of Glasgow, Glasgow, UK

**Key messages**

- In causal inference, the adjustment of covariates should typically be based on causal assumptions rather than purely informed by data
- The primary causal effect of interest is the extent to which the outcome is changed when the exposure is intervened on
- To minimise bias in estimating causal effect in observational studies, generally confounders should be adjusted but mediators and colliders should not be

This paper outlines the principles on the use of regression adjustment for causal inference in observational studies, and discusses when to adjust or not adjust for a covariate

## Introduction

Causal inference identifies and quantifies the effect of an exposure (the hypothetical cause) to an outcome. While randomised controlled trial design is the preferred method for establishing and measuring causal effects, they are costly and not always feasible. Observational studies can be used for causal inference, but confounding is a key limitation. Typically, a confounder is a variable that causes the exposure and outcome. If left unaccounted for, confounders distort the estimate away from the true causal effect. Regression analysis is a common technique to analyse observational data, partly because of its ability to adjust for confounders by including those confounders as independent variables in the regression model. However, knowing when a variable should be adjusted is not always clear.

**Figure 1:**
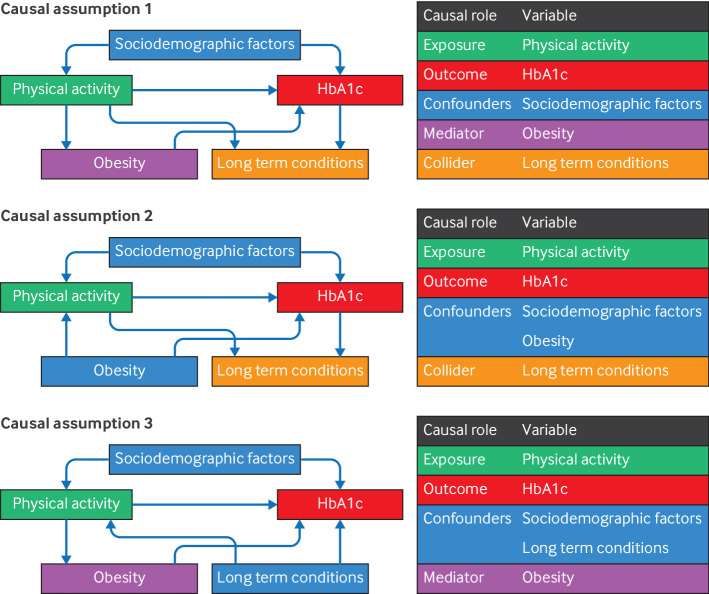
Direct acyclic graphs on the potential causal hypotheses for the association between physical activity and glycated haemoglobin (HbA1c). Boxes represent variables of causal interest and arrows denote causal directions. In this research question where physical activity is the exposure and HbA1c is the outcome, sociodemographic factors (age, sex, and education) are assumed to be confounders. In causal assumption 1, obesity is assumed to be a mediator while long term conditions are assumed to be a collider. Causal assumptions 2 and 3 assume obesity and long term conditions to be confounders, respectively. The plausibility of these assumptions determines whether sociodemographic factors, obesity, and long term conditions should be adjusted. Various long term conditions (such as circulatory and chronic kidney disease) can be caused by lower physical activity and high levels of HbA1c, and thus can be a possible mediator. Therefore, regression estimates between physical activity and HbA1c when adjusted for long term conditions (model 3, table 1) might be biased. However, long term conditions can also be argued to affect physical activity (eg, by limiting mobility ^16^) and HbA1c levels (eg, anaemia can increase HbA1c levels^17^), whereby causal assumption 3 might also be possible—in this case, the results from model 3 would be valid instead

Consider an example study to examine whether meeting the World Health Organization's (WHO) physical activity recommendation (exposure) could influence glycated haemoglobin (HbA1c; outcome), a biomarker for diabetes,^1^ using the Health Survey for England 2016 with 3013 respondents with valid data.^2^ In addition to the exposure and outcome variables, sociodemographic factors (age, sex, education), obesity, and long term conditions are also likely to have causal roles in this association. Causal assumptions can be visualised in a direct acyclic graph. Figure 1 shows direct acyclic graphs for the example study on physical activity and HbA1c.

## Adjustment of confounders

When conducting a simple linear regression (ie, unadjusted), we are comparing the mean value of the outcome (in this case, HbA1c) between exposed and unexposed individuals (ie, those meeting the physical activity recommendations or not) without considering other variables. Model 0 (table 1) shows the finding of a simple linear regression analysis. It suggests that people who met WHO's physical activity recommendation on average had a HbA1c level of 2.7 mmol/mol lower than those who did not meet WHO's physical activity recommendation. In model 0, we ignore any differences between the people who met and who did not meet the WHO recommendation on physical activity. If the data had been from a well conducted randomised controlled trial, such analysis should still uncover a causal estimate as confounding is eliminated through study design.^3^ However, because the current data were from an observational study, this estimate is subject to confounding bias. We do not know if the difference in HbA1c is due to physical activity or confounding (eg, ageing reduces physical activity and increases HbA1c levels simultaneously). Literature has suggested that people who were male, younger, and more educated are more likely to have more physical activity.^4^ Sex, age, and socioeconomic status are also likely to influence HbA1c levels.^5^

**Table 1:** Association between physical activity and glycated haemoglobin (HbA1c) in different regression models

Characteristic	Adjusted mean difference (95% CI)	P value
Model 0: unadjusted	−2.7 (−3.4 to −2.1)	<0.001
Model 1A: adjusted for age, sex, and education	−1.8 (−2.4 to −1.2)	<0.001
Model 1B: model 1A with age and education on sex specific P-splines	−1.7 (−2.3 to −1.2)	<0.001
Model 2: model 1B plus obesity	−1.5 (−2.1 to −0.96)	<0.001
Model 3: model 1B plus long term conditions	−1.4 (−2.0 to −0.85)	<0.001

CI, Confidence interval.

Regression adjustment is a common way in dealing with confounding bias. Adjusting for a confounder will eliminate any bias it causes in the adjusted exposure-outcome association, if the confounder is correctly measured and modelled. In this example, age, sex, and education are such confounders, because they are assumed to be causes of higher physical activity and lower HbA1c levels, as shown by the arrows in figure 1 (causal assumption 1). For model 1A (table 1), the association between physical activity and HbA1c was attenuated after adjusting for sex, age, and education compared with model 0. However, removing confounding bias based on regression adjustment relies on various untestable assumptions, such as accurately identifying and measuring confounders^6^ and correctly modelling confounder-outcome associations.^7^ In this example, model 1A assumes that HbA1c is linearly related to age and education and assumes no interaction between these variables, which may not be true. If age and education are non-linearly related to HbA1c and such associations were dependent on sex, the variables can be modelled using sex specific P-splines instead.^8^ Model 1B, which modelled age using sex specific P-splines, showed the results where the exposure-outcome association was further attenuated. This estimate should be relatively robust if causal assumption 1 in figure 1 is true and no confounders are missing. In practice, all the measured confounders identified on the basis of subject knowledge should be adjusted, but it is impossible to prove that there are no missing confounders in an observational study design.

## Adjustment of mediators

Some epidemiological studies appear to adjust for as many relevant variables as possible, without any clear justification. This practice should be avoided because some variables might not be confounders, but may be mediators and colliders of the exposure and outcome. Mediators are intermediate variables between the exposure and outcome, and should not be adjusted if the analysis aims to uncover the total effect of the exposure.^9^ Obesity, for instance, can be a mediator between physical activity and HbA1c because physical activity can reduce body weight^10^ and obesity is a risk factor for higher levels of HbA1c.^11^ If we adjust our regression model for obesity (model 2, table 1), the regression estimate between physical activity and HbA1c is further attenuated, but this could underestimate the total effect from physical activity to HbA1c if obesity is a mediator (ie, causal assumption 1 is true). However, the model 2 estimate would be valid if obesity is a confounder (causal assumption 2, figure 1). Similar to the identification of confounders, subject knowledge should inform whether a variable should be treated as a confounder or a mediator.

## Adjustment of collider

Colliders are the common effect of the exposure and the outcome. Adjusting for collider will bias estimates of the exposure-outcome association away from the causal effect of the exposure to the outcome.^12^ In this example study of physical activity and HbA1c levels, long term conditions could be argued as a possible collider (causal assumption 1, figure 1). If causal assumption 1 is indeed true, findings from model 1 should be more accurate. However, it is not always clear whether a variable is a collider or a confounder. figure 1

## If causality is unclear

In practice, the decision of regression adjustment for causal inference needs to be guided by the research question and subject knowledge. Even in causal machine learning methods, causal roles of variables have to be assumed.^12^

An attenuation of regression estimate after the adjustment of a variable could indicate that adjusted variable to be a confounder, a mediator, a collider, or even none of the above.^13^ In other words, estimate attenuates after adjustment does not necessarily imply any causal roles. The identification of confounders, therefore, cannot rely on only data. In practice, numerous variables could be related to the exposure and outcomes, and identifying which are confounders may be difficult. Graphical methods based on the so-called backdoor criterion could be a more systematic way to identify a set of adjustment variables under a specific causal assumption.^14^ Specifically, the backdoor criterion suggests adjusting for all variables that point towards the exposure (hence the term "backdoor") but that also creates a path to the outcome. It is implemented by a web tool^15^ and is particularly useful when known confounders are not measured directly but the causes of the unmeasured confounders are.

In the example study on the causal effect of physical activity on HbA1c levels, the causal roles of obesity and long term conditions can be argued as either confounders or mediator/collider, and this would affect whether they should be adjusted. This question has no definitive answer, especially because this study has a cross sectional design where the time sequence of these variables is not established. However, longitudinal design with multiple time points, and triangulation based on alternative study design (eg, mendelian randomisation), may be useful. In addition, sensitivity analysis could also help to show whether results are robust to causal assumptions. In this example, if causal assumption 1 is believed to be more likely, researchers may pick model 1 as their primary analysis and supplement with results from models 2 and 3, acknowledging that causal assumptions 2 and 3 are also possible. If the results from models contradict, researchers should be more careful because the assumptions on the causal roles of obesity and long term conditions could be critical to the research question. However, if all models provide similar conclusions, as in this case, the causal roles of obesity or long term conditions may be less important.

In summary, generally confounders, but not mediators or colliders, should be adjusted in causal inference. The identification of confounders needs to be based on subject knowledge and is not always clear, but the use of systematic methods and triangulation could help. In any case, the selection of adjustment variables should be thought out carefully and justified.
